# Using machine learning methods to determine a typology of patients with HIV-HCV infection to be treated with antivirals

**DOI:** 10.1371/journal.pone.0227188

**Published:** 2020-01-10

**Authors:** Antonio Rivero-Juárez, David Guijo-Rubio, Francisco Tellez, Rosario Palacios, Dolores Merino, Juan Macías, Juan Carlos Fernández, Pedro Antonio Gutiérrez, Antonio Rivero, César Hervás-Martínez

**Affiliations:** 1 Unidad de Enfermedades Infecciosas, Hospital Universitario Reina Sofía de Córdoba, Instituto Maimónides de Investigación Biomédica de Córdoba, Universidad de Córdoba, Córdoba, España; 2 Departamento de Informática y Análisis Numérico, Universidad de Córdoba, Córdoba, España; 3 Unidad de Enfermedades Infecciosas, Hospital Universitario de Puerto Real, Cádiz, España; 4 Unidad de Enfermedades Infecciosas, Hospital Juan Ramón Jiménez e Infanta Elena de Huelva, Huelva, España; 5 Unidad de Enfermedades Infecciosas, Hospital Universitario Virgen de la Victoria, Complejo Hospitalario Provincial de Málaga, Málaga, España; 6 Unidad de Enfermedades Infecciosas, Hospital Universitario de Valme, Instituto de Biomedicina de Sevilla, Sevilla, España; Centers for Disease Control and Prevention, UNITED STATES

## Abstract

Several European countries have established criteria for prioritising initiation of treatment in patients infected with the hepatitis C virus (HCV) by grouping patients according to clinical characteristics. Based on neural network techniques, our objective was to identify those factors for HIV/HCV co-infected patients (to which clinicians have given careful consideration before treatment uptake) that have not being included among the prioritisation criteria. This study was based on the Spanish HERACLES cohort (NCT02511496) (April-September 2015, 2940 patients) and involved application of different neural network models with different basis functions (product-unit, sigmoid unit and radial basis function neural networks) for automatic classification of patients for treatment. An evolutionary algorithm was used to determine the architecture and estimate the coefficients of the model. This machine learning methodology found that radial basis neural networks provided a very simple model in terms of the number of patient characteristics to be considered by the classifier (in this case, six), returning a good overall classification accuracy of 0.767 and a minimum sensitivity (for the classification of the minority class, *untreated* patients) of 0.550. Finally, the area under the *ROC* curve was 0.802, which proved to be exceptional. The parsimony of the model makes it especially attractive, using just eight connections. The independent variable “recent PWID” is compulsory due to its importance. The simplicity of the model means that it is possible to analyse the relationship between patient characteristics and the probability of belonging to the *treated* group.

## Introduction

Chronic hepatitis C virus infection (HCV) is a major cause of cirrhosis, liver transplantation and liver-related deaths worldwide [1]. Since HCV and HIV share routes of transmission, it is common to find that HIV-infected patients are also infected with HCV [2], which carries the worst prognosis in these patients due to its faster progression and comorbidities [3, 4]. Hence, treatment uptake in this population is mandatory. In the last few years, direct-acting antiviral drugs (DAAs) with high cure rates (defined as sustained virological response) have become available for the treatment of HCV infection [5]. Even though there is a strong recommendation for universal treatment of this disease [6, 7], due to the high numbers of patients waiting for treatment, the scientific societies and health authorities have established various prioritisation criteria for initiating therapy based on achieving maximum survival and clinical benefits for the patient. The implementation of this strategy and the commitment of clinicians to these recommendations have not so far been evaluated. Identifying patient-related variables that could limit treatment uptake in HIV/HCV co-infected patients, even when the prioritisation criteria for treatment are fulfilled, is an important issue.

Multilayer perceptron (MLP) artificial neural networks (ANN) [8] have been widely used in this field to model nonlinear functions for classification. Several studies have demonstrated that the methodology is appropriate in this context: Wang *et al*. [9] successfully applied the ANN methodology to predict virological response to therapy from HIV genotype. Resino *et al*. [10] studied an ANN trained to predict significant fibrosis among HIV/HCV co-infected patients using clinical data derived from peripheral blood, concluding that the ANN technique was a helpful tool in clinical practice for guiding therapeutic decisions in HIV/HCV co-infected patients. Lamer *et al*. [11] demonstrated the use of ANNs trained using evolutionary computation to predict R5, X4 and R5X4 HIV-1 co-receptor usage; their results indicated the identification of R5X4 viruses with a predictive accuracy of 75.5%. Pradhan and Sahu [12] presented a new MLP network that used seven different patient characteristics as inputs (age, sex, weight, HB, CD3, CD8 and TB) to classify the HIV/AIDS-infected and non-infected status of individuals. In short, the literature shows that this methodology has already been successfully applied in the field of HIV prediction and obtained good performance [13].

Feedforward neural networks, in which the information moves in a forward direction, are the commonest and simplest type of ANN [14]. Our study proposal included three ANNs that differed according to the basis function used: product unit neural network (PUNN) [15], sigmoid unit neural network (SUNN) [8], and finally, the radial basis function neural network (RBFNN) [16]. All these methods have been widely used in biomedicine since 1990 and are still in use today: see [17–19] for RBFNN, [20–22] for MLP or SUNN and [23, 24] for PUNN. Finally, all these ANN models have been proven to be universal approximators [8]. Moreover, there are various applications of evolutionary neural network models in biomedicine. Vukicevic *et al*. presented an evolutionary algorithm to train ANNs to predict the outcome of surgery for choledocholithiasis [25]. Cruz-Ramírez *et al*. used a multi-objective evolutionary algorithm to train RBFNNs to predict patient survival after liver transplantation [26]. Dorado-Moreno *et al*. two approaches in combination, a cost-sensitive evolutionary ordinal ANN and an ordinal over-sampling technique, to tackle the same problem [27].

The main objective of this study was to develop an empirical and parsimonious classification model to treat/not treat HIV/HCV-infected patients with antiretrovirals, trying to maximise overall accuracy, to achieve a good classification for the minority class (*untreated* patients) and to obtain a good performance in all the possible classification thresholds. Spain, which provides universal and free health care access, established different criteria in April 2015 for the initiation and prioritization of HCV treatment, which are known as the Spanish National Strategy for HCV treatment. This strategy recognised different scenarios based on the disease severity (such as liver fibrosis stage and extrahepatic manifestations), comorbidities, epidemiology (such as risk of transmission population or women wishing to be pregnant), etc. [10]. The application of this strategy has had an evident beneficial impact on short-term treatment uptake [11]. Nevertheless, clinical, epidemiological and geographic factors associated with lower treatment odds have not been evaluated. Understanding patient factors associated with being untreated for HCV would help in supporting extra efforts in those patients in order to eliminate HCV in the coming years. In this sense, a large number of experimental tests were carried out with several basis functions associated with different neural network types. The secondary objective was to find the simplest possible model able to analyse the influence of patient characteristics on the probability of belonging to the *treated* group.

## Materials and methods

### Resource and setting

The patients considered were part of the HERACLES cohort. This prospective observational cohort included HIV-infected patients with active chronic HCV infection in follow-up at 19 reference centres in Andalusia (clinicaltrials.gov identification: NCT02511496). Active chronic HCV infection was defined as detectable HCV RNA in serum or plasma for 6 months or more. The cohort was set up in March 2015 with the main objective of evaluating the HCV treatment rate among included patients. The population included in this cohort represented 99.9% of HIV-infected individuals in follow-up in Andalusia. Patients included in the cohort were followed-up every three months according to clinical practice. The time period of this analysis was 2 years.

### Criteria for initiation of HCV treatment

Treatment was initiated in each individual in accordance with the prioritisation criteria established in Spain’s national strategic plan for HCV treatment. This strategic plan recognises different scenarios and criteria based on disease severity (such as liver fibrosis stage and extrahepatic manifestations), comorbidities, and epidemiology (such as population transmission risk or women hoping to become pregnant). Nevertheless, the final decision to initiate HCV therapy was taken by the clinician in charge. Patients who initiated therapy were classified as those who i) met the criteria, or ii) did not meet the criteria, depending on whether they did or did not satisfy the criteria for HCV treatment set out in the strategic plan.

### Variable collection and definition

The following variables were included and recorded: age, gender, route of transmission of HIV and HCV, HCV genotype, liver fibrosis stage, history of HCV therapy (treatment-naïve, Peg-IFN/RBV-treated patients, DAAs + Peg-IFN/RBV-treated patients), comorbidities, presence of active major psychiatric disorders, recent drug abuse, opioid substitution therapy (OST) use, convictions, and adherence to clinical visits. People who inject drugs (PWIDs) were categorised as lifetime PWID (people who had injected drugs at some point but there is no current OST use or drug abuse), OST-PWID (OST use, but no drug use in the last 3 months) and recent PWID (evidence of drug consumption in the previous 3 months). Liver transient elastography by FibroScan (FibroScan; Echosens, Paris) was used for liver stiffness measurements (LSM) and grading and staging of liver fibrosis. Liver fibrosis stages were defined as follows: i) *F*0 − *F*1 ≡ *LSM* < 7.2 *kPa*; ii) *F*2 ≡ 7.2 ≤ *LSM* ≤ 8.9 *kPa*; iii) *F*3 ≡ 9 ≤ *LSM* ≤ 14.5 *kPa*; and iv) *F*4 ≡ *LSM* ≥ 14.6 *kPa*.

### ANN models

The problem considered in this study was to predict the need for treatment of patients co-infected with HIV/HCV. To estimate the model, a training set of *N*_*T*_ samples was required, $\left(x_{i},y_{i}\right),i=1\ldots N_{T},x_{i}\in R^{d},y_{i}\in \left\{0,1\right\}$, where *d* is the number of inputs of the model and *y*_*i*_ represents a binary variable coding the need of treatment (*y*_*i*_ = 1) or the absence of this need (*y*_*i*_ = 0). Nonlinear functions were applied to solve the problem, specifically the following artificial neural networks (ANN): product unit neural networks (PUNN) [28], sigmoid unit neural network (SUNN) [29], and radial basis function neural network (RBFNN) [16].

The differences between the proposed basis functions are as follows: PUNN models are highly versatile for implementing high-order functions, retaining the properties of a universal approximator while using only a small number of neurons with multiplicative rather than additive units [30]. SUNN models use sigmoid transfer functions for hidden layer nodes. This is the most widely used type of neural network because of its ability to approximate any continuous function with sufficient accuracy. Finally, RBFNN models approximate underlying functions by using a linear combination of semiparametric nonlinear functions, such as Gaussians. This kind of function has two main advantages: the simplicity of its structure and the speed of the learning algorithms it employs. None of these models requires a large number of neurons to solve certain problems [31], which makes them reasonable choices to apply to this problem.

To train the ANN models, an evolutionary algorithm inspired on that developed by Angeline *et al*. [32] and extended afterwards [28, 33] was used, with the purpose of estimating the parameters and the architecture of the ANNs. The use of evolutionary learning for designing these models dates back to the 1990s (see [34] for an initial review and [35] for a more recent one). Much work has been done during this period, leaving many different approaches and working models [36–39].

In this way, evolutionary computation has been used to learn both the architecture and the connections and weights of the neural network [28]. The main advantage of evolutionary computation is that it performs a global exploration of the search space to avoid becoming trapped in local minima, which is often the case with local search procedures.

### Population and characteristics

This study was based on the Spanish HERACLES cohort (NCT02511496) (April-September 2015), which included 2940 HIV/HCV co-infected patients with the characteristics shown in Table 1.

**Table 1:** Characteristics of the Spanish HERACLES cohort (NCT02511496).

Description	Variable	Values	Occurrences	Percentage
Met the Spanish criteria plan	*X*_1_	No	1287	43.77%
		Yes	1653	56.22%
PWID Category	*X*_2_	Lifetime PWID	2169	73.78%
	*X*_3_	OST PWID	339	11.53%
	*X*_4_	Recent PWID	47	1.60%
	*X*_5_	Never PWID	385	13.10%
Presented major psychiatric disorders	*X*_6_	No	2886	98.16%
		Yes	54	1.84%
Been in jail	*X*_7_	No	2823	96.02%
		Yes	117	3.98%
Previous treatment experience	*X*_8_	Naïve to therapy	2053	69.83%
	*X*_9_	With Peg-IFN/RBV	725	24.66%
	*X*_10_	With DAAs/Peg-IFN/RBV	162	5.51%
Liver fibrosis	*X*_11_	Stage F0-F1	898	30.54%
		Stage F2	475	16.16%
		Stage F3	787	26.77%
		Stage F4	780	26.53%
Gender	*X*_12_	Female	491	16.70%
		Male	2449	83.30%
Age	*X*_13_	Continuous variable	min	18
			max	76
			mean	48.95
HCV genotype	*X*_14_	Genotype 1	1741	59.22%
	*X*_15_	Genotype 2	27	0.92%
	*X*_16_	Genotype 3	484	16.46%
	*X*_17_	Genotype 4	688	23.40%
Received therapy for HCV infection	*y*	No	988	33.60%
		Yes	1952	66.40%

At the end of follow-up, of those 1952 patients who received therapy against HCV chronic infection, 1348 (69.0%) met the criteria of Spain’s strategic plan for HCV treatment, and 604 did not (31.0%). And of the 988 patients who did not receive therapy, 305 (30.8%) met the criteria for receiving therapy according to the strategic plan for HCV treatment, and 683 did not (69.2%).

At the end of follow-up, of the 1952 patients who received treatment for HCV chronic infection, 1348 (69.0%) fulfilled the criteria for HCV treatment laid down in Spain’s strategic plan and 604 did not (31.0%). Of the 988 patients who did not receive treatment, 305 (30.8%) met the criteria for receiving therapy according to the strategic plan for HCV treatment and 683 did not (69.2%).

### Experimental design

Before training the model, the input variables were scaled in the range [1, 2] for PUNN models to prevent input values close to zero, which produces large values in the case of negative exponents. The upper boundary was chosen to avoid substantial changes on the outputs when weights and exponents are high. For SUNN models, the inputs were scaled in the range [0.1, 0.9] to avoid saturation in the sigmoid basis function when weights are very high. Finally, in the case of the RBFNN models, the inputs were scaled in the range [−1, 1] since these functions are symmetric on the origin. The following Equation shows an example of the scale of input *X*_*i*_ for SUNN models:

$$\begin{array}{c} X_{i}^{*}=\frac{X_{i}-\min\left(X_{i}\right)}{\max\left(X_{i}\right)-\min\left(X_{i}\right)}\left(0.9-0.1\right)+0.1. \end{array}$$ (1)

For the experimental design, the holdout procedure was used: the training set size was 75% of the whole dataset, while the remaining 25% was used for the generalisation set.

The performance of each model was evaluated according to accuracy (also known as Correct Classification Rate, *CCR*), minimum sensitivity (*MS*), area under the *ROC* curve (*AUC*), and number of connections (#*conn*), the latter being used to determine the parsimony of the model. The *CCR* and *MS* were obtained from the confusion matrix, *CM*:

$$\begin{array}{c} CM=\{n_{ij};\sum_{i,j}^{J}n_{ij}=N\}, \end{array}$$ (2)

where *J* is the number of classes (two in this case), *N* is the number of training or testing patterns, and *n*_*ij*_ represents the number of times the patterns are predicted to belong to class *j* when they really belong to class *i*. The first two measures of classifier performance, *CCR* and *MS*, were calculated from the confusion matrix, while the *AUC* was obtained from the predicted probabilities:

- The *CCR* measure is given by the expression $CCR=\frac{1}{N}\sum_{j=1}^{J}n_{jj}$, where *n*_*jj*_ is the number of patterns from the *j*-th class that are correctly classified in that class. In other words, *CCR* is the sum of the elements belonging to the diagonal of the confusion matrix divided by *N*. *CCR* is a value between 0 and 1, where 0 means that none of the instances have been classified correctly, while 1 involves that there were no errors for any instance.
- *MS* is the minimum value of the sensitivities for each class [40], which is defined as *MS* = *min*(*S*_1_, *S*_2_), where *S*_*j*_ is the sensitivity for class *j*, i.e. $S_{j}=100\frac{n_{j}}{N_{j}}$, *n*_*j*_ being the number of instances correctly classified for class *j* and *N*_*j*_ being the total number of instances for class *j*. *MS* is a value between 0 and 1, where 0 means that one class was completely misclassified, while 1 means there were no errors for any class.
- *AUC* is the area under the *ROC* curve, which is a common technique to compare the performance of two or more binary classifiers and is especially common in medical decision making [41]. The *ROC* curve is a graphical plot that illustrates the relative trade-offs between the costs and benefits of a classifier, enabling visual comparison of different classifiers. The *AUC* is used to make numerical comparisons. *AUC* is a value between 0 and 1, where 0 means that all the predictions made were incorrect, while 1 means that all instances were correctly classified.

The evolutionary algorithm was run using the following parameters. In the case of product units, the weights between the input layer and hidden layer were initialised in the range [−1, 1] and those between the hidden layer and output layer in the range [−5, 5]. In the case of the sigmoidal units and radial basis functions, both weights were initialised in the range [−5, 5]. The population size was 2940, randomly split into two datasets: 2193 instances were used for training and the remaining 747 instances were used for the generalisation set. Since the evolutionary algorithm is a stochastic method, the algorithm was repeated 30 times for 600 generations, with a different random seed for each run. In addition, the number of nodes and connections to be created or deleted fell within the range [1, 2]. Finally, the minimum number of hidden nodes, the maximum number of hidden nodes in the initialisation phase and the maximum number of hidden nodes in the whole evolutionary process were set at 1, 2, and 4, respectively. All these values were selected following a 5-fold cross-validation on the training set, and the remaining values were obtained from Hervás-Martínez *et al*. [42].

### Results

The performance of each of the proposed techniques was measured according to test *CCR*, test *MS*, test *AUC* #*conn*. The performance of the best model, including the set of independent variables finally considered for the model, is shown in Table 2. Based on these results and focusing on the highest *AUC* values obtained, the RBFNN model stands out with an *AUC* of 0.802, indicating that it is good at separating *treated* from *untreated* patients. Apart from the *AUC*, the RBFNN also returned competitive values for the other performance metrics, with scores of 0.550 for *MS*, indicating that the minority class was correctly classified, and 0.767 for the *CCR*, which is the global performance of the classifier. It should be borne in mind however that the *CCR* is not advisable for imbalanced datasets (in our case, 1952 *treated* and 988 *untreated* patients). Finally, the main advantage of this technique is the low number of connections used, 8.

**Table 2:** Values of test *CCR*, *MS* and *AUC*, together with #*conn*, using PUNN, SUNN and RBFNN. The independent variables selected for the best model are also shown. The best result is highlighted in **bold** face; the second best result is shown in *italics*.

	Mean±SD			
Model	*CCR*	*MS*	*AUC*	#*conn*
PUNN (17-1,2,4-1)	*0.767 ± 0.004*	*0.559 ± 0.012*	*0.794 ± 0.003*	*9.46 ± 1.57*
SUNN (17-1,2,4-1)	0.762 ± 0.006	**0.563 ± 0.014**	0.793 ± 0.002	14.10 ± 1.63
RBFNN (17-1,2,4-1)	**0.768 ± 0.004**	0.550 ± 0.008	**0.795 ± 0.005**	**7.36 ± 1.83**
RBFNN2 (16-1,2,4-1)	0.456 ± 0.127	0.014 ± 0.019	0.483 ± 0.028	7.33 ± 2.17
	Best Model			
Model	*CCR*	*MS*	*AUC*	#*conn*
PUNN (17-1,2,4-1)	**0.771**	**0.579**	*0.801*	*11*
SUNN (17-1,2,4-1)	**0.771**	0.545	0.799	19
RBFNN (17-1,2,4-1)	*0.767*	*0.550*	**0.802**	**8**
RBFNN2 (16-1,2,4-1)	0.514	0.000	0.573	9
Independent variables considered:				
PUNN (17-1,2,4-1)	*X*_2_, *X*_4_, *X*_5_, *X*_9_, *X*_11_, *X*_13_, *X*_14_, *X*_17_			
SUNN (17-1,2,4-1)	*X*_2_, *X*_3_, *X*_4_, *X*_6_, *X*_7_, *X*_9_, *X*_10_, *X*_11_, *X*_13_			
RBFNN (17-1,2,4-1)	**X**_**3**_, **X**_**4**_, **X**_**8**_, **X**_**11**_, **X**_**13**_, **X**_**16**_			
*RBFNN*2 (16-1,2,4-1)	*X*_1_, *X*_9_, *X*_10_, *X*_11_, *X*_12_, *X*_13_, *X*_14_			

The PUNN technique also achieved a good performance, yielding a score of 0.801 for the *AUC* and 0.579 for *MS*, but using a higher number of connections, 11. The last technique, SUNN, gave the worst *AUC* and *MS* performance and used the highest number of connections, 19.

In order to assess the quality of the ANN models, a comparison against Support Vector Machines (SVMs) [43] was carried out. In this way, a 5-fold cross-validation method optimising the *AUC* measure, was run to select the best value for the penalty of the error (*C*) and for the RBF kernel coefficient (*γ*), both chosen within the range {10^−4^, 10^−3^, …10^2^}. The results obtained were 0.755 in terms of *CCR* and 0.716 for *AUC*, being clearly worse than the ones obtained by the models in Table 2, whereas in terms of *MS*, it led to 0.603, slightly better than the *MS* obtained by the ANN models.

Attention is drawn to the importance of input variable *X*_4_ (“recent PWIDs”) among the classifiers used, since it was included in all the best models and its non-inclusion in the RBFNN model (called *RBFNN*2) reduced the mean *AUC* from 0.795 ± 0.005 to 0.483 ± 0.028 and the mean *MS* from 0.550 ± 0.008 to 0.014 ± 0.019, making it a trivial classifier that classifies most instances in one class.

Taking into account the mean values obtained, it may be concluded that the best technique is the RBFNN, since it achieved the best results for *AUC*, #*conn* and *CCR* and a reasonably good performance for *MS*. It is also worth noting that the use of just six input variables makes the model easy to interpret, easy to implement and requires little training time, while the rest of the techniques need more than 6 input variables.

**Fig 1:**
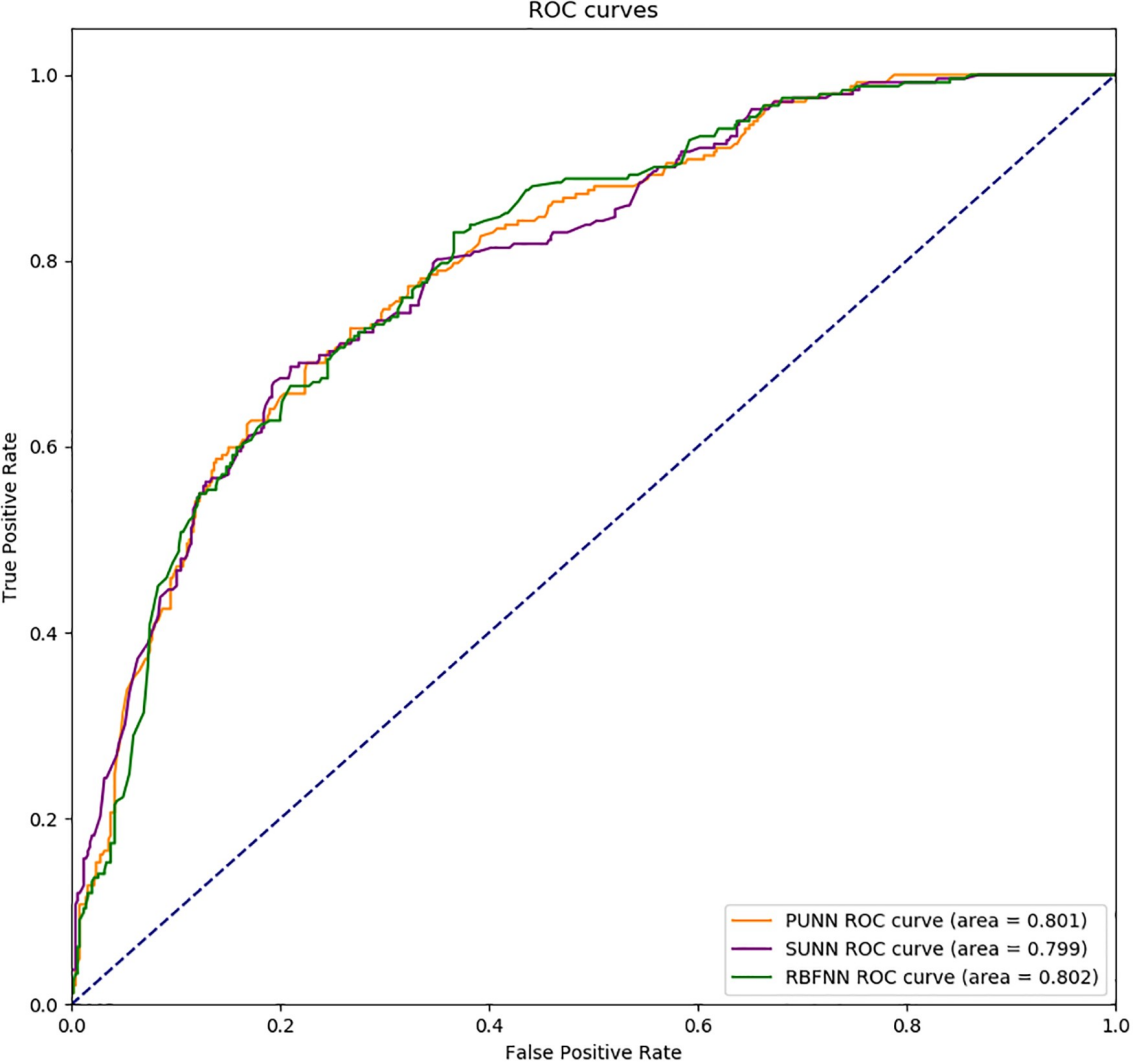
*ROC* curve for the three models proposed.

The *ROC* curves for the three methods proposed are shown in Fig 1. The *ROC* curve provides a graphical display of true positives (*TPR*) and false positives (*FPR*) on the *x*− and *y*− axes, respectively, where *TPR* is equivalent to *sensitivity*, and *FPR* is equal to 1 − *specificity* for varying cut-off points of test probability values. Although the performance of all the models was competitive, it can be seen that the RBFNN provided the best results.

Furthermore, it could be thought that the application of a preprocessing technique to reduce the dimensionality of the input variables would be of interest. However, it would make the models deal with two additional disadvantages: a important lost of interpretability, since the new input variables are combinations of the original ones, and a possible lost of performance, due to the need of more robust and accurate information. In this sense, Principal Components Analysis (PCA) [44] was run, concluding that 90% of the variance was explained by using 12 variables, which is greater than the number of independent variables used by all the models shown in Table 2. In this way, the RBFNN was run following the same experimental design but considering this set of principal components as input variables. The average results of the 30 runs are 0.752 ± 0.007 in terms of *CCR*, 0.550 ± 0.026 in terms of *MS* and 0.767 ± 0.008 in terms of *AUC*, which are worse than the results obtained by all the models with the original datasets. Furthermore, regarding the best model, it achieved the following values: 0.750, 0.537 and 0.782 for *CCR*, *MS* and *AUC*, respectively. These are also worse than the results obtained by the best models with the original dataset.

To consider the statistical significance of differences between means (*CCR*, *MS*, *AUC* and #*conn*) for each ANN topology (SUNN, PUNN and RBFNN), the non-parametric Kolmogorov-Smirnov (K-S) test for normality was used with *α* = 0.05, to evaluate whether *CCR*, *MS*, *AUC* and #*conn* followed a normal distribution. Remember that all ANN models were run 30 times with different seeds. As can be seen from the results in Table 3, a normal distribution can be assumed because the critical levels, *p*-values, were greater than 0.05 in most cases. One-way ANOVA was used to determine the best methodology (in terms of *CCR*, *MS*, *AUC* and #*conn*). The results of the ANOVA analysis showed that the effect of the methodology was statistically significant at a significance level of 5% (see the first row of Table 4). This test determined that there were significant differences between the results found by the different methods, and multiple comparison tests were then carried out on the *CCR*, *MS*, *AUC* and #*conn* values to rank the different methods. The Levene test [45] was used to evaluate equality of variances, followed by the Tukey test [46], since the variances were equal (for all *CCR*, *MS*, *AUC* and #*conn*), to rank the different methods.

**Table 3:** P-values of the Kolmogorov-Smirnov test applied to the generalisation set for *CCR*, *MS*, *AUC* and #*conn*.

Variable	PUNN	SUNN	RBFNN
*CCR*	0.138	0.200	0.003
*MS*	0.034	0.002	< 0.001
*AUC*	0.200	< 0.001	0.028
#*conn*	0.029	0.007	0.023

**Table 4:** P-values of Snedecor’s F ANOVA I test ordered means for the multiple comparison Tukey test when considering *CCR*, *MS*, *AUC* and #*conn* for the models obtained.

	*CCR* *	*MS* *
F (*p*-values)	< 0.001	< 0.001
Ranking of averages	*μ*_*PUNN*_ ≤ *μ*_*RBFNN*_: *p* = 0.218	*μ*_*PUNN*_ ≤ *μ*_*SUNN*_: *p* = 0.368
	*μ*_*SUNN*_ < *μ*_*RBFNN*_: *p* < 0.001	*μ*_*RBFNN*_ ≤ *μ*_*PUNN*_: *p* = 0.022
	*μ*_*SUNN*_ < *μ*_*PUNN*_: *p* < 0.001	*μ*_*RBFNN*_ ≤ *μ*_*SUNN*_: *p* < 0.001
	*AUC*	#*conn* *
F (*p*-values)	0.221	< 0.001
Ranking of averages	No significant differences	*μ*_*PUNN*_ < *μ*_*SUNN*_: *p* < 0.001
		*μ*_*RBFNN*_ < *μ*_*SUNN*_: *p* < 0.001
		*μ*_*RBFNN*_ < *μ*_*PUNN*_: *p* < 0.001

(*) Significant differences were found for *α* = 0.05.

As can be concluded from Table 4, RBFNN obtained statistically significant results for both *CCR* and #*conn*, although for *MS*, the results were worse than those obtained with the other models. The best model in terms of *MS* was SUNN. Finally, with respect to the *AUC*, there were no significant differences, although the best results were obtained by RBFNN.

The equations for the different models are provided in S1 Table. Based on these equations, it can be concluded that the RBFNN model was not particularly complex, using just eight connections and six independent variables, achieving a high performance. Table 5 shows the main characteristics of this model, together with the variables considered, *CCR*, *MS*, *AUC* and the confusion matrices (*CM*) on both the training and generalisation sets.

**Table 5:** Main characteristics of the RBFNN model.

Variables	*X*_3_: OST PWID	
	*X*_4_: recent PWID	
	*X*_8_: Naïve to therapy	
	*X*_11_: Liver fibrosis (1, 2, 3, 4)	
	*X*_13_: Age	
	*X*_16_: Genotype 3	
	Training	Generalisation
*CCR*	0.757	0.767
*MS*	0.522	0.550
*AUC*	0.801	0.802
*CM*	$\left(\begin{array}{cc} 1270 & 177\\ 357 & 389 \end{array}\right)$	$\left(\begin{array}{cc} 440 & 65\\ 109 & 133 \end{array}\right)$

The table shows a reasonable *CCR* in the generalisation set, taking into account the small number of independent variables. The *MS* indicates that the sensitivity for each class is good enough, bearing in mind the imbalance between them. The *AUC* shows that our model has good discriminatory power between patients in the *treated* and *untreated* classes. The confusion matrices show the distribution of errors and the number of correctly classified patterns.

**Fig 2:**
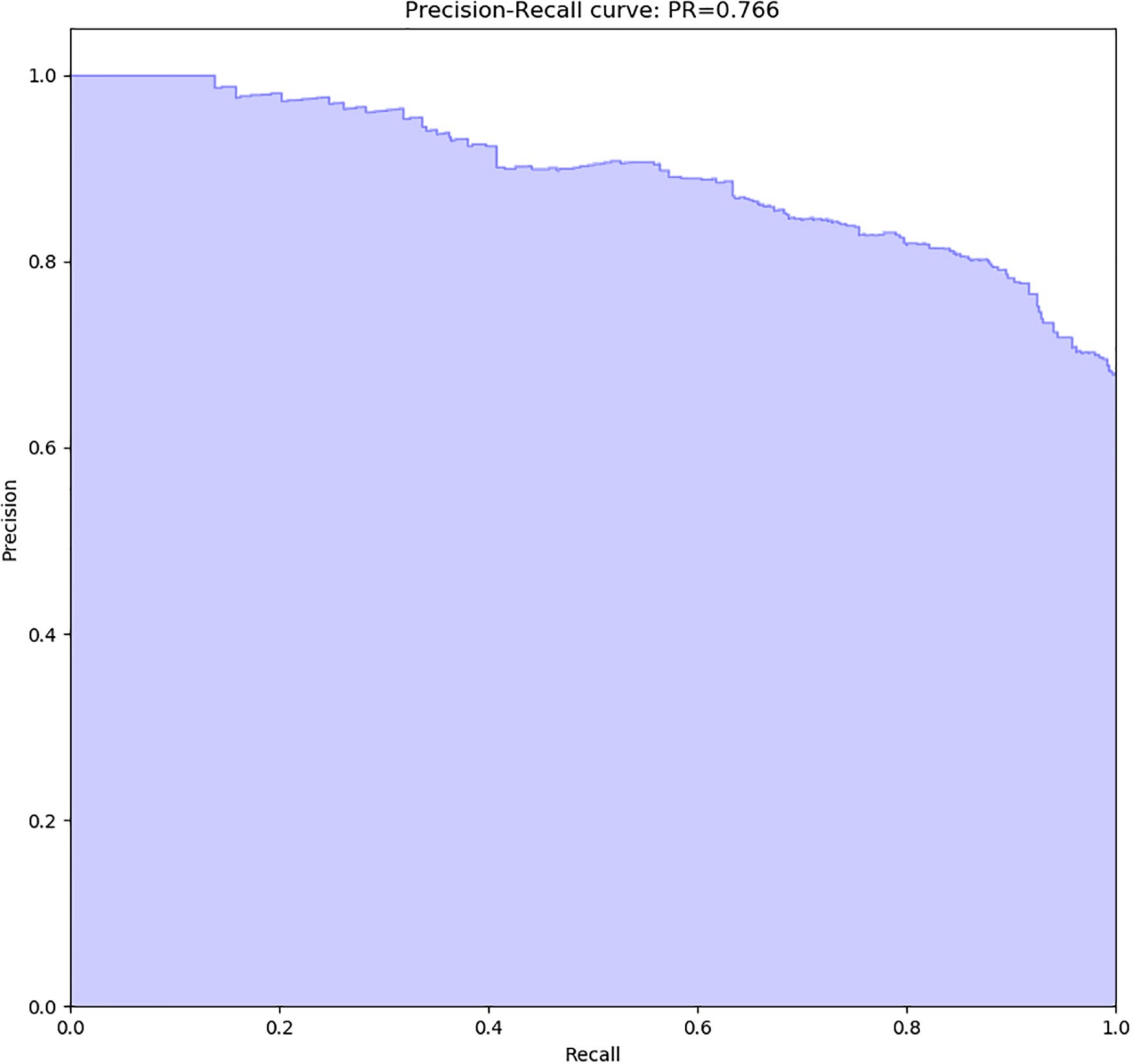
Precision-Recall curve for the best model obtained.

The Precision-Recall metric (*PR*) was calculated for this best model. The precision-recall metric is a useful measure of success of prediction when the classes are very imbalanced. A high *AUC* represents both high precision and high recall. In this case, *PR* was 0.766. The precision-recall curve is also shown in Fig 2 for evaluating the trade-off between precision and recall for different thresholds.

## Discussion

Three main points can be outlined for the best model:

1. The output function of the RBFNN model is a linear combination of radial basis functions (see Table 5 and S1 Table) with a positive coefficient of 8.957, which means that the higher the value of the basis function, the greater the probability of being *treated*.
2. The importance of the independent variable *X*_4_ (“Recent PWID”) is worthy of mention. When this variable is left out of the model (see Table 2), accuracy decreases from 0.767 to 0.514, the minimum sensitivity decreases from 0.550 to 0.000, meaning that the model is not a good classifier of any of the patterns belonging to the minority set (*untreated* patients) and the *AUC* curve decreases from 0.802 to 0.573. Inclusion of this variable should therefore be mandatory because of its importance.
3. The parsimony of the model, in other words, the small number of independent variables, is what makes it especially attractive: it is not necessary to obtain further information from the patient, it reduces the time needed to obtain the same prediction accuracy, and it minimises the likelihood of incurring in information errors.

## Conclusion

Application of a machine-learning methodology enabled us to identify variables associated with lower uptake of HCV treatment. The variable “Recent PWID” was identified as the main limiting factor related to the absence of treatment uptake, even when the prioritisation criteria were met. This is a critical variable in the sense that absence of treatment uptake in this population would involve a significant risk of HCV dissemination and the appearance of outbreaks. A recent HIV and HCV outbreak associated with injection-drug use of oxymorphone in the United States is a clear example of the importance of this point [47]. Recent PWIDs should therefore be reconsidered as a priority population for implementation of HCV treatment in order to minimise the risk of community-acquired HCV infection and maximise the impact of therapy, leading to the objective of eliminating HCV in the future. The use of radial basis functions neural networks, very simple models with regards to the number of patient characteristics to be considered by the classifier, might be a useful tool for drawing up or modifying strategic plans when tackling different diseases and, more specifically in the present case, for maximising the impact of therapy. Indeed, Intelligent Network DisRuption Analysis (INDRA) has been employed as a targeted strategy for the efficient interruption of hepatitis C transmission among PWIDs [48]. In our opinion, its use in clinical decision making in infectious diseases should be expanded with a view to optimising recommendations for treatment and prevention strategies.

## Supporting information

S1 Table Best models obtained for the different methodologies. (PDF)
