# Supplementary material for: Using machine learning methods to determine a typology of patients with HIV-HCV infection to be treated with antivirals
Source: PLoS One. 2020 Jan 10;15(1):e0227188. doi: 10.1371/journal.pone.0227188 (PMC6953863; doi:10.1371/journal.pone.0227188)
Supplement: S1 Table — (PDF) [file pone.0227188.s001.pdf]

| | |
|---|---|
| PUNN | $g_{(T)} = -1.476 + 7.874 \cdot (X_2^{0.123} \cdot X_4^{-0.410} \cdot X_5^{0.155} \cdot X_9^{0.287} \cdot X_{10}^{0.219} \cdot X_{11}^{0.526} \cdot X_{13}^{0.116} \cdot X_{14}^{0.085} \cdot X_{17}^{0.108})$ |
| SUNN | $g_{(T)} = 0.649 + \frac{3.037}{1+e^{(2.750-0.423X_2-3.389X_4-0.123X_7-5.000X_8+3.846X_{10}+2.037X_{13})}} - \frac{4.994}{1+e^{(-0.847+0.268X_2+0.702X_3+5.000X_4+0.272X_6-0.064X_7+0.890X_9+1.148X_{10}-4.090X_{11})}}$ |
| RBFNN | $g_{(T)} = -3.710 + 8.957 \cdot \left( e^{\left( \frac{-0.5 \cdot \left( (X_3-0.425)^2+(X_4-0.101)^2+(X_8-0.273)^2+(X_{11}-0.848)^2+(X_{13}-0.654)^2+(X_{16}-0.442)^2 \right)^{0.5}}{0.752} \right)} \right)^2$ |
